# Transformation of autophagic SQSTM1 droplets to SQSTM1-dependent P-bodies

**DOI:** 10.1080/15548627.2024.2340413

**Published:** 2024-04-10

**Authors:** Evelina Valionyte, Elizabeth R. Barrow, Chris R. Baxter, Sharon Herath, Shouqing Luo

**Affiliations:** Peninsula Medical School, Faculty of Health, University of Plymouth, Research Way, Plymouth, Devon, UK

**Keywords:** Autophagy, NLRP3 inflammasome, P-bodies, PYCARD, SQSTM1

## Abstract

SQSTM1/p62 droplets play crucial roles in droplets-based macroautophagy/autophagy including selective autophagy and bulk autophagy. We observed that under several stress milieus, SQSTM1 droplets entirely colocalize with P-body markers, and these stress-induced SQSTM1 droplets contain mRNAs. We thus determined that under certain stress conditions, autophagic SQSTM1 droplets are converted to a type of enlarged P-bodies, designated SQSTM1/p62-dependent P-bodies (pd-PBs). Stress-enhanced SQSTM1 droplet formation drives the nucleation of pd-PBs through the interaction between SQSTM1 and the RNA-binding protein DDX6. Furthermore, pd-PBs sequester PYCARD, facilitating the assembly of NLRP3 inflammasomes, and in turn induce inflammation-related cytotoxicity. Our study suggests that under stress settings, autophagic SQSTM1 droplets are transformed to pd-PBs, underlining a critical role of SQSTM1 in P-body condensation.

SQSTM1 droplets play vital roles in selective autophagy and bulk autophagy. SQSTM1 droplet formation is driven by the binding of polyubiquitinated proteins, a process critical for cargo selection and autophagosome biogenesis. However, it was unknown whether SQSTM1 could exist in a differential droplet format. RNP granules including P-bodies (PBs) and stress granules (SGs) are important to maintain cellular homeostasis in stress conditions. Persistent RNP granules have been widely reported to associate with amyotrophic lateral sclerosis/ALS and frontotemporal dementia/FTD. Concurrently, SQSTM1 mutations may also be associated with the amyotrophic lateral sclerosis-frontotemporal dementia spectrum.

We were initially interested in the fact that stress conditions, including puromycin and lipopolysaccharide (LPS), significantly enhance SQSTM1 droplet size. According to the experiments with fluorescence recovery after photobleaching, puromycin-induced SQSTM1 droplets are more dynamic than those under basal conditions. We aimed to identify the components of puromycin-enlarged SQSTM1 droplets. To this end, we adopted the BioID2 approach to identify SQSTM1-proximity proteins [1]. RNA-binding proteins (RBPs), such as DDX6, and LSM14A, are among the identified proteins. Cycloheximide stabilizes polysomes, resulting in the disassembly of cytoplasmic RNA granules like SGs and PBs. Cycloheximide treatment entirely negates the enlargement of SQSTM1 droplets induced by puromycin, suggesting that mRNA could be a constituent of stress-enlarged SQSTM1 droplets. In situ hybridization by oligo(dT)30, which complement the mRNA poly(A) tail, showed that puromycin-induced SQSTM1 droplets are positive for mRNAs. Thus, stress-induced SQSTM1 droplets would represent a type of RNP granules. Indeed, puromycin-enlarged SQSTM1 droplets entirely colocalize with the markers of PBs, including DCP1A, DDX6, LSM14A, and EDC4, but not SG markers including G3BP1, G3BP2 and CAPRIN1. Furthermore, probing of oligo(dT)30 demonstrated that SQSTM1 droplets with the P-body marker EDC4 contain mRNAs.

SQSTM1 knockdown effectively abolishes the levels of PBs, marked with EDC4, under proteotoxic stress. Consistently, EDC4-marked PBs are almost lost in *SQSTM1* knockout cells. Thus, SQSTM1 is required for the formation of stress-enlarged PBs, termed SQSTM1/p62-dependent (pd-PBs). We asked whether the critical RBP components of PBs are needed for stress-induced SQSTM1 droplet formation. Ablation of EDC4 or LSM14A blocks stress-induced SQSTM1 droplet formation. The reciprocal roles of SQSTM1 and PBs’ crucial components in stress-induced PB nucleation and stress-enlarged SQSTM1 droplet formation indicate that stress-enlarged SQSTM1 droplets represent stress-induced PBs, or pd-PBs, strengthening the concept that autophagic SQSTM1 droplets are transformed to pd-PBs under stress conditions.

The PB1 domain at the SQSTM1 N terminus is required for SQSTM1 oligomerization, and the UBA domain at the SQSTM1 C terminus binds to polyubiquitinated proteins, which are critical for SQSTM1 clustering. In SQSTM1-deficient cells, the SQSTM1 N terminus (amino acids 1–256) fails to form pd-PBs, unlike full-length SQSTM1. This implies that the SQSTM1 C terminus is necessary for the formation of pd-PBs. SQSTM1 droplet formation, which depends on polyubiquitin binding, would be a driving force for the nucleation of pd-PBs. Indeed, the chemical inhibitor TAK-243 that blocks protein polyubiquitination, stops the formation of puromycin-induced pd-PBs. Polyubiquitinated protein binding to SQSTM1 drives the latter’s droplet formation. With both polyubiquitin chains and SQSTM1, DDX6 undergoes rapid condensation and gelation in vitro. The reconstituted system suggests that SQSTM1 droplet formation elicits GFP-DDX6 phase condensation, supporting the hypothesis that enlarged pd-PB formation is driven by enhanced SQSTM1 droplet formation. Collectively, we concluded that enhanced SQSTM1 droplet condensation, induced by stresses including proteotoxicity, facilitates the nucleation of pd-PBs.

Stimulation by LPS and oxidation causes a strong induction of pd-PBs in macrophages. We investigated SQSTM1-binding proteins in macrophages by SQSTM1 immunoprecipitation and identified PYCARD/ASC as a binding partner. We were intrigued to examine if PYCARD is sequestered by pd-PBs, because PYCARD plays a critical role in inflammasome formation. LPS and nigericin, which initiate a priming signal and activation signal, respectively, induce NLPR3 inflammasome formation. Whereas SQSTM1 droplets do not colocalize with PYCARD under basal conditions, LPS + nigericin treatment causes the recruitment of PYCARD by pd-PBs in macrophages. LPS-induced pd-PBs modestly colocalize with PYCARD; however, nigericin leads to the profound colocalization of PYCARD with pd-PBs. NLPR3 and CASP1 are also assembled into pd-PBs in macrophages under LPS + nigericin treatment. This finding suggests that pd-PBs function as platforms for the formation of inflammasomes. We knocked down EDC4, DDX6, or LSM14A to eliminate pd-PBs, given that these RBPs are critical for the formation of PBs. Knockdown of EDC4, LSM14A or DDX6 ameliorates CASP1 activation. Furthermore, knockdown of EDC4, DDX6, or LSM14A reduces LPS + nigericin-induced NLRP3 inflammasome cytotoxicity. Moreover, knockdown of these P-body components significantly reduces the production of IL1B. Our data show that pd-PBs sequester PYCARD, thereby facilitating the assembly of NLRP3 inflammasomes. Transformation of SQSTM1 bodies to pd-PBs upon endotoxin stress acts as a stress response to induce inflammasome activation.
